# Optimizing the Functional and Safety Properties of a Marine Allergen: Maillard-Induced Conjugation of Chitosan and Saccharides Attenuates the Allergenicity of Turbot (*Scophthalmus maximus*) Parvalbumin

**DOI:** 10.3390/foods15071259

**Published:** 2026-04-07

**Authors:** Linda Dzadu, Qi’an Han, Sheng Yin, Manman Liu, Shiwen Han, Huilian Che

**Affiliations:** 1 Key Laboratory of Food Nutrition and Health Evaluation Technology, State Administration for Market Regulation, College of Food Science and Nutritional Engineering, China Agricultural University, Beijing 100083, China; 2 School of Food & Health, Beijing Technology and Business University, Beijing 100048, China

**Keywords:** parvalbumin, fish allergy, chitosan, Maillard reaction, allergenicity reduction, epitope masking, antioxidant activity, immunomodulation

## Abstract

Fish allergy, mainly caused by Parvalbumin (PV), is a worldwide health issue with few effective mitigation options. This study investigated Maillard conjugation using chitosan (CS) and various saccharides to modify the structural, functional, and allergenic properties of turbot (*Scophthalmus maximus*) PV. Structural analyses, including SDS-PAGE, Western blotting, FTIR spectroscopy, and Circular dichroism, confirmed successful conjugation and significant changes in secondary structure, including decreases in α-helical content and increases in β-sheet and random-coil fractions. Glycation significantly boosted antioxidant activity, with total phenolic content (TPC) increasing up to 10.3 times and DPPH radical scavenging reaching 74.5% in the CS–xylose–PV conjugate (CXTPV). Indirect ELISA revealed notable (*p* < 0.05), sugar-dependent reductions in IgE-binding capacity, with reductions of up to approximately 72% for CXTPV. RBL-2H3 cell assays showed decreased β-hexosaminidase release (about 75% reduction), lowered IL-6 secretion, and strong inhibition of IL-4 production, indicating reduced allergenic potential and immune regulation. CXTPV demonstrated the best overall performance. These findings suggest that CS–saccharide Maillard conjugation is an effective approach for creating hypoallergenic marine ingredients with improved bioactive properties.

## 1. Introduction

Food allergies are an increasing public health concern, with fish allergy among the most prevalent and severe immunoglobulin E (IgE)-mediated reactions [1]. In most fish species, including turbot (*Scophthalmus maximus*), the primary allergen is Parvalbumin (PV), a small, acidic calcium-binding protein with a low-molecular-weight (MW) of 10–14 kDa and an acidic isoelectric point (pI 3.0–5.0) [2,3]. Its highly stable 3D structure comprises three EF-hand motifs, with the CD and EF loops serving as critical calcium-binding sites that maintain the protein’s globular conformation [4,5]. These structural features directly affect PV’s allergenicity because many IgE-reactive epitopes are conformational and calcium-dependent [6]. Removing calcium ions greatly reduces IgE binding by disrupting the protein’s tertiary structure [6,7]. Furthermore, linear IgE epitopes have been identified across different fish species using peptide microarray and immunoinformatics methods [3,6,7], including specific immunodominant regions such as residues 64–78 in grouper and 19–33 in salmon [8]. Studies comparing α and β parvalbumin isoforms have also reported differences in reactivity [6,9]. This variation in epitope reactivity explains why some fish species cross-react, while others cause species-specific sensitization, illustrating the complexity in diagnosing and managing fish allergy [6,10].

The Maillard Reaction (MR), also known as protein glycation, is a non-enzymatic reaction that occurs at elevated temperatures between the free amino groups of proteins and reducing sugars [11,12]. This complex cascade of chemical reactions, often classified into initial, intermediate, and final stages, is fundamental to the development of color, flavor, and aroma in many thermally processed foods [13]. This reaction, which plays a key role in food chemistry, significantly alters protein structure and immunoreactivity [14,15].

To utilize this reaction for allergen-specific mitigation of allergy, we chose chitosan (CS) as a biopolymer support [16]. CS, a cationic biopolymer obtained through alkaline N-deacetylation of chitin, features a linear polysaccharide chain with randomly distributed beta-(1-4)-linked D-glucosamine and N-acetyl-D-glucosamine units [16,17]. Its MW ranges from 50 to 190 kDa, with a degree of deacetylation of approximately 75%. This range enables the formation of stable glycation products via the MR, while preserving the solubility required for functional allergenicity tests [3,17].

Traditional heat treatment induces non-specific structural changes, making it difficult to consistently and irreversibly eliminate PV allergenicity, thereby leaving core allergenic epitopes intact [7,9]. In contrast, targeted glycation via the MR has emerged as a popular and effective technological approach to producing hypoallergenic foods by masking epitopes and reducing protein immunogenicity [7,18]. When combined with CS’s inherent biocompatibility and functional versatility, these conjugates demonstrate enhanced antioxidant activity and improved stability, positioning them as promising ingredients for both functional foods and advanced biomedical applications [3,7,16,17]. We recommend that a rationally designed macromolecular conjugation approach be the primary paradigm. This study aims to confirm that a Maillard-induced conjugate between PV and a CS–saccharide complex is more effective. The main hypothesis is that this structural modification will reduce allergenic potential by steric hindrance and electrostatic disruption of typical IgE-binding epitopes. This mechanism is strongly consistent with recent studies demonstrating inhibition of IgG/IgE binding through direct sugar glycation of epitopes and conformational changes [3,19,20]. Specifically, research on β-PV isoforms indicates that these structural modifications effectively reduce immunoreactivity by masking key allergen epitopes [3,20]. Various studies have shown that it effectively reduces PV immunoreactivity [3,21]. Concurrently, the process will generate advanced functional Maillard reaction products (MRPs) with rigorous antioxidant activity [11,13]. The compelling rationale of this dual-objective approach is the unique ability of site-specific covalent glycation to permanently mask critical IgE-binding epitopes through steric hindrance and conformational disruption, a decisive mechanistic advantage that harnesses CS–saccharide chemistry to generate advanced MRPs with potent antioxidant and functional properties. This study aims to validate the enhanced efficacy of Maillard-induced conjugation between turbot PV and a CS–saccharide complex, evidenced by a synergistic decrease in allergenic potential through efficient steric and conformational masking of epitopes, as well as the production of novel MRPs with strong antioxidant activity. Through rigorous comparative analysis of the structural, functional, and immunological properties of the conjugates relative to a thermally processed PV control, this work establishes a rational and robust framework for developing low-allergenic PV with enhanced bioactivity.

## 2. Materials and Methods

### 2.1. Materials

Fresh turbot (*Scophthalmus maximus*) was purchased from a local seafood market in Beijing, China, and transported on ice to the lab within 30 min. Chemicals such as CS (medium MW: 50–190 kDa, ≥95% deacetylation, cat no. C804726-100g) were purchased from Macklin Inc, Shanghai, China. D-(+)-glucose, D-maltose, D-lactose, D-xylose, D-sucrose, Bradford reagent, tris(hydroxymethyl)aminomethane hydrochloride (Tris-HCl; pH 8.0), glycine, EDTA, and other analytical-grade substances were obtained from Sigma-Aldrich in Shanghai, China. Monoclonal Anti-PV IgG antibody from mouse sera immunized with fish PV (cat no. P3088, Millipore Sigma Aldrich, Burlington, MA, USA), and HRP-conjugated anti-human IgE antibodies (cat no. AP175, Millipore Sigma, Burlington, MA, USA), HRP-conjugated mouse anti-mouse IgG (cat no. ab97240, Abcam, Shanghai, China). Rat basophilic leukemia-2H3 (RBL-2H3) cells were acquired from the Chinese Academy of Medical Sciences in Beijing, China. Human serum pools from individuals with confirmed fish allergy (based on clinical history) were used for IgE-binding analysis. These sera were aliquoted and stored at −80 °C until use. Enzyme-linked immunosorbent assay kits for -hexosaminidase, interleukin-4 (IL-4; cat. no. 70-EK304/2-96), and interleukin-6 (IL-6; cat. no. 70-EK306/3-96) were acquired from MultiSciences Biotech Co., Ltd., Hangzhou, China. TMB two-component substrate solution (cat. no. PR1210) was from Solarbio, Beijing, China, and Bovine Serum Albumin (cat. no. SRE0098) was obtained from Sigma-Aldrich, Burlington, MA, USA. Triton X-100 (cat. no. T8787) from Sigma-Aldrich, Burlington, MA, USA. All other reagents were of analytical grade unless otherwise specified.

#### Human Serum Samples

The study’s experimental protocol and informed consent forms were reviewed and approved by the Human Research Ethics Committee of China Agricultural University (ethics approval number: CAUHR-20251007). All aspects of the study were conducted under this ethics committee’s oversight. All volunteers involved in the study were recruited from within China Agricultural University. Volunteers ranged in age from 18 to 60 years and included no gender restrictions. A total of 21 patients diagnosed with food allergies or allergic rhinitis, along with volunteers who self-reported food allergies or allergic rhinitis, were recruited. Blood samples were collected at the East Campus Hospital of China Agricultural University. Serum samples were drawn into clotting tubes, left at room temperature for 2 h, then centrifuged at 3000 rpm for 15 min. The separated serum was collected, packaged, and stored at −80 °C for future analysis of antibody and cytokine levels.

### 2.2. Preparation of Fish Fillet and PV Extraction

Fresh turbot (*Scophthalmus maximus*) was transported on ice, and fish fillets were extracted using a slightly modified method from Ma J and colleagues [22]. Briefly, 5 g of fish fillet was homogenized with 30 mL of ice-cold Tris extraction buffer (0.1 mol/L, pH 7.4, containing 0.5 mmol/L glycine, with or without 0.1 mmol/L dithiothreitol) at an initial solid-to-solvent ratio of 1:6 (*w*/*v*). To optimize protein yield and maintain structural stability, the extraction conditions were systematically tested by varying temperature (0 °C, 4 °C, 8 °C, and 12 °C), extraction time (6, 12, 24, 36, and 48 h), and solid-to-solvent ratios (1:2, 1:6, 1:10, and 1:14, *w*/*v*). Typically, baseline conditions were set at 4 °C for 12 h with a 1:6 (*w*/*v*) ratio, and one parameter was varied at a time to identify the most efficient extraction conditions. After extraction, the homogenates were centrifuged at 4000 rpm for 30 min at 4 °C, and the supernatants were dialyzed against 0.1 mol/L phosphate-buffered saline (PBS, pH 7.4) for 24 h. The resulting PV extract was stored at −20 °C for further analysis.

### 2.3. Purification of PV

PV was purified from the crude extract using a sequential ammonium sulfate precipitation method adapted from Swoboda and colleagues [23] with slight modifications. The crude extract was first brought to 65% saturation with ammonium sulfate, and the precipitate was discarded after centrifugation (15,000 *g* for 30 min at 4 °C). The supernatant was then adjusted to 90% saturation, and the resulting pellet, containing the enriched PV fraction, was collected. The pellet was dissolved in 20 mM Tris-HCl (pH 8.0) with 1 mM EDTA and dialyzed against 50 mM sodium bicarbonate buffer (pH 8.3) for 24–36 h to remove salts. The dialyzed sample was concentrated using a 3.5 kDa MW cutoff centrifugal concentrator. SDS-PAGE and Western blot (WB) were employed to assess the purity of the extracted PV. Protein concentration was measured (4.226 mg/mL) before storage at −80 °C. For electrophoresis, 15 µL of a 1 mg/mL sample was loaded into each well of a 15% Bis-Tris gel (12-well, GenScript cat no. M00677, Nanjing, China). Separation was conducted using 2-(N-morpholino) ethanesulfonic acid (MES) buffer at a steady 200 V for about 20–30 min, according to the manufacturer’s instructions.

### 2.4. Preparation of CS–PV–Sugar Conjugates and Control

The glycation of PV with CS was carried out using a controlled-heating method based on MR conjugation principles, with slight modifications [24]. Five sugars, D-maltose, D-xylose, D-lactose, D-glucose, and D-sucrose, were chosen for glycation with CS and turbot PV. A CS stock solution was first prepared at 1% (*w*/*v*) in 1% (*v*/*v*) acetic acid, while individual 1% (*w*/*v*) sugar solutions were made in 0.01 M PBS, pH 7.4. Each sugar solution was then mixed with the CS stock at a 1:1 mass ratio under constant stirring for 30 min at room temperature. Purified PV was dissolved in PBS to a concentration of 2 mg/mL. For conjugation, each CS–sugar mixture was combined with the PV solution in a 1:1:1 mass ratio, and the pH of all final reaction mixtures was adjusted to 7.4. The glycation reaction proceeded by lyophilizing for 48 h, followed by incubation under controlled conditions (using an incubator) at 60 °C for 72 h, resulting in the corresponding conjugates: CS–xylose–turbot–PV (CXTPV), CS–glucose–turbot PV (CGTPV), CS–sucrose–turbot PV (CSTPV), CS–maltose–turbot PV (CMTPV), and CS–lactose–turbot PV (CLTPV). A native control (NTPV) was prepared in parallel by heating a PV-PBS mixture without CS or sugar under the same conditions. All reactions were cooled in an ice bath for 20 min to terminate. Finally, the conjugates and the control were freeze-dried and stored at −20 °C.

### 2.5. Characterization of Structural Changes

The browning index, as an indicator of MR, was estimated by measuring absorbance at 420 nm using the standard method [6]. Absorbance at 420 nm in diluted crude extract samples (200 μL) was measured using a microplate reader (Supermax, Shanghai, China).

#### 2.5.1. SDS-PAGE Analysis

Protein samples (1 mg/mL) were mixed with 2X Laemmli loading buffer and heated at 95 °C for 5 min, according to Tai and colleagues [3], with some modifications. Fifteen microliters of the samples were loaded into each well of a 15% Bis-Tris gel (12-well, GenScript). Separation was conducted using 2-(N-morpholino) ethanesulfonic acid (MES) buffer at a steady 200 V for about 20–30 min, according to the manufacturer’s instructions. The gels were stained with Coomassie Brilliant Blue to visualize the protein bands.

#### 2.5.2. WB Analysis

This analysis followed the method of Wu and colleagues [21], with minor modifications. Proteins separated by SDS-PAGE (15 µL of a 1 mg/mL sample loaded into each well of a 15% 12-well Bis-Tris gel). The gels were transferred onto nitrocellulose or PVDF membranes. These membranes were blocked with Bovine Serum Albumin (BSA) and then incubated with anti-PV IgG antibodies derived from mouse sera immunized against fish PV to verify the identity and antigenicity of the protein bands. Subsequently, membranes were treated with an HRP-conjugated mouse anti-mouse IgG. The signals were developed using an enhanced chemiluminescent (ECL) HRP substrate kit (cat no. WBKLS0100, Millipore Sigma, Burlington, MA, USA) and visualized.

#### 2.5.3. Circular Dichroism (CD) Spectroscopy

The secondary structure of the protein samples was analyzed using CD spectroscopy [25]. Measurements were conducted on an Applied Photophysics CD Spectrometer (UK) in the far-UV range (195–250 nm) with a nitrogen purge. Proteins were diluted to 0.2 mg/mL before analysis. The CD spectra were processed using Jasco software (ver. 2.5 CFR, Tokyo, Japan), and the relative amounts of secondary structural elements were quantitatively assessed using the BeStSel (Beta Structure Selection) platform.

#### 2.5.4. Fourier Transform Infrared (FTIR) Spectroscopy

To assess changes in protein structure, FTIR spectroscopy was performed on the glycated and control samples, following established methods for spectral acquisition and analysis of the Amide 1 region [26,27]. Freeze-dried sample powders were thoroughly mixed with potassium bromide (KBr) at approximately 1:100 (sample: KBr) and pressed into transparent pellets under vacuum. Spectra were recorded in transmission mode (4000–400 cm^−1^, 4 cm^−1^ resolution, 64 scans). Secondary structure was quantified by deconvoluting the amide I region (1600–1700 cm^−1^). Spectral deconvolution and initial estimation of secondary-structure components were performed using the BeStSel algorithm. The deconvoluted amide I bands were subsequently fitted and refined using Prism software, with sub-bands assigned as follows: ∼1690 cm^−1^ (β-turn), ∼1675 cm^−1^ (α-helix), ∼1655 cm^−1^ (random coil), and ∼1640 cm^−1^ (β-sheet). The quality-corrected R^2^ values for all fitted curves exceeded 0.99.

#### 2.5.5. Analysis of Free Amino Acid Content

The analysis of free amino acids was performed using liquid chromatography-mass spectrometry (LC-MS) according to an established method with minor modifications [28]. An aliquot of the mixed sample was homogenized with 0.9 mL of 50% acetonitrile solution for 5 min. The resulting mixture was vortexed and then centrifuged at 12,000 rpm for 10 min. The supernatant was filtered through a 0.22 μm microporous membrane before LC-MS analysis. Chromatographic separation was achieved using a Waters BEH Amide column (2.1 × 100 mm, 1.7 μm) maintained at 35 °C. The mobile phase was delivered at a constant flow rate of 0.35 mL/min, and an injection volume of 2.0 μL was used. Detection was performed using an electrospray ionization source in positive-ion mode.

### 2.6. Analyses of Bioactive Compounds and Antioxidant Activity

#### 2.6.1. Total Phenolic Content (TPC)

TPC was determined using the method of Martinez and colleagues [29]. Aliquots of 0.5 mL of the samples were mixed with 2.5 mL of Folin–Ciocalteu reagent and 2 mL of NaCO3 (7.5%; *w*/*v*). The mixture was heated at 45 °C for 15 min in a water bath in the dark, then left to stand for 30 min before absorbance was measured at 765 nm. The total phenolic content is expressed as gallic acid equivalents (GAE)/100 g of fresh matter based on a standard curve of gallic acid (0–100 mg/kg).

#### 2.6.2. 2,2-Diphenyl-1-Picrylhydrazyl (DPPH) Radical Scavenging Capacity

The DPPH radical-scavenging capacity of MRPs and the control was evaluated using the method of Du and colleagues [30], with minor adjustments. The DPPH working solution is obtained by dissolving DPPH powder in ethanol. Twenty microliters of the samples were combined with 180 μL of the DPPH working solution (0.2 mM) in a 96-well microplate, then shaken and incubated in the dark at room temperature for 30 min. Subsequently, the absorbance of the mixed solution was measured at 517 nm using a microplate reader, with L-ascorbic acid as a positive control. The DPPH free radical scavenging rate is calculated as follows:

$$\left(\%\right)=\frac{\left[absorbance\;of\;blank-\left\{absorbance\;of\;tested\;sample-absorbance\;of\;control\right\}\right]}{\;absorbance\;of\;blank}\;\times 100$$

### 2.7. Indirect ELISA

CS-glycated samples and NTPV were quantified using an indirect enzyme-linked immunosorbent assay (ELISA) with allergen-specific IgE, adapted from methods used in food allergy research [3] with minor modifications. For coating, each well was treated with 100 μL of the respective PV sample (untreated PV, glycated PV, and NTPV at 2 μg/mL) in coating buffer and incubated at 4 °C overnight. This ensured that different wells contained distinct PV variants, depending on the experimental group and reaction conditions. After five washes with washing buffer (0.05% Tween 20 in PBS), the plate was blocked with 200 μL per well of blocking buffer (5% BSA in PBS) at 37 °C for 1.5 h. Pooled serum from turbot-allergic patients as diluted in 1% BSA/PBS. To evaluate specific IgE binding capacity, the plates were incubated with patient sera for 2 h at 37 °C. Following a washing step to remove unbound IgE antibodies, the plates were incubated with a goat polyclonal IgE-specific HRP-conjugated secondary antibody for 1 h at 37 °C. A subsequent washing step was then performed to remove any unbound secondary antibody before detection. The plates were washed thoroughly, and the reaction was developed using 3,3′,5,5′-Tetramethylbenzidine (TMB) substrate. Color development was stopped using 50 μL of 2 M H_2_SO_4_, and absorbance was measured at 450 nm. A native control group labeled “PV (untreated)” was free of CS, sugar, and heating. It was added to serve as a baseline for the experiment. The IgE binding capacity was calculated as:

$$\%IgE=\frac{\left(ODsample-ODblank\right)}{ODcontrol-ODblank}\;\times 100$$

### 2.8. Mediator and Cytokine Release

#### 2.8.1. Cell Culture

The RBL-2H3 cells were grown according to the method of Song and Li [31] with minor modifications. Initially, the cells were cultured in Dulbecco’s Modified Eagle’s Medium (DMEM) supplemented with 10% Fetal Bovine Serum (FBS), streptomycin (100 μg/mL), and penicillin (100 U/mL). Next, the cells were incubated in a 5% CO2/95% air atmosphere at 37 °C.

#### 2.8.2. β-Hexosaminidase Release

The RBL-2H3 cell degranulation assay was performed according to established protocols, with modifications to enhance sensitization [32]. RBL-2H3 cells (5 × 10^5^ cells/mL, 200 μL per well) were plated in a 96-well plate and grown to confluence. Twenty-four hours later, the cells were washed with PBS to remove residual growth factors and non-adherent cells. Then, 10 μL of diluted serum in minimum essential medium without PBS was added to each well for passive sensitization. Afterward, the cells were washed three times with Tyrode’s buffer (containing 135 mM NaCl, 5 mM KCl, 1 mM MgCl_2_, 1 mM CaCl_2_, 20 mM HEPES, 5.6 mM glucose, and 0.05% BSA, pH 7.4). Fifty microliters of samples (100 ng/mL) dissolved in Tyrode’s buffer were added, and incubation proceeded for 45 min at 37 °C. Positive controls received 1% Triton X-100, while negative controls received only Tyrode’s buffer. The reaction was halted on ice, and 30 μL of supernatant was collected. Lastly, 20 μL of sodium carbonate buffer was added, and absorbance was read at 405 nm using a microplate reader. The β-hexosaminidase release levels were calculated as:

$$\left(\%\right)=\frac{\left(absorbance\;of\;tested\;sample-absorbance\;of\;blank\right)}{\left(absorbance\;of\;control\;(triton\;X)-absorbance\;of\;blank\right)}\;\times 100$$

#### 2.8.3. Cytokine Analysis

Glycated samples and NTPV were centrifuged at 3000× *g* for 10 min at 4 °C to pellet debris and granules. IL-4 and IL-6 levels in the supernatant were measured by ELISA, following the instructions of Friedel J and colleagues [33]. A control group, labeled low serum medium (LSM), used low-serum medium instead of the samples’ medium.

### 2.9. Statistical Analysis

Data (*n* = 3, *n* = 6) were analyzed using one-way ANOVA with Tukey’s post hoc test (GraphPad Prism v9.0). Results presented as mean ± SD; *p* < 0.05 is considered statistically significant.

## 3. Results

### 3.1. Extraction, Purification, and Identification of PV

**Figure 1:**
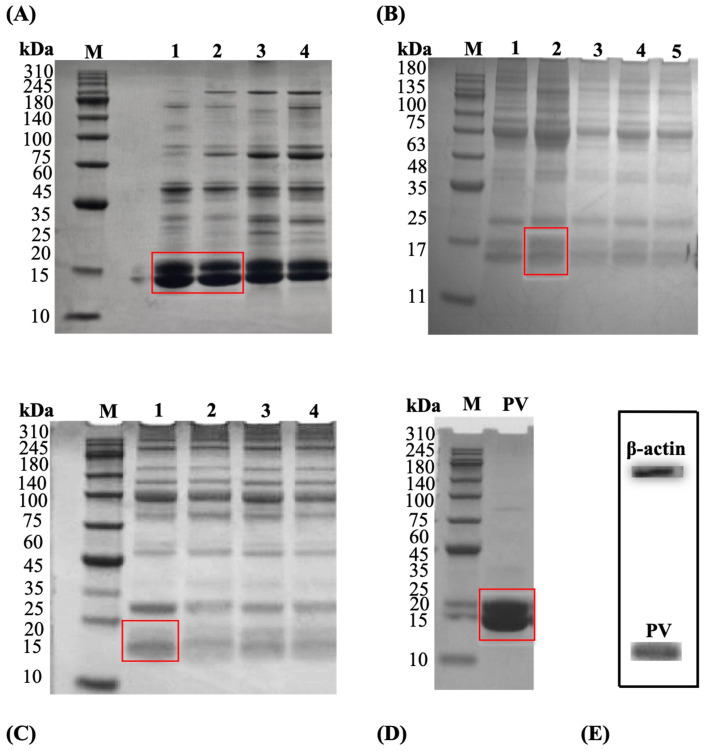
Optimization of turbot PV extraction from fish muscle. (**A**) SDS-PAGE of extracts obtained at different temperatures (0, 4, 8, and 12 °C) for 12 h with a 1:6 (*w*/*v*) solvent-to-sample ratio. (**B**) SDS-PAGE of extracts obtained at different extraction times (6, 12, 24, 36, and 48 h) at 4 °C with a 1:6 (*w*/*v*) ratio. (**C**) SDS-PAGE of extracts obtained at different solvent-to-sample ratios (1:6, 1:10, 1:14, and 1:18 *w*/*v*) at 4 °C for 12 h. (**D**) Quantification of PV band intensity (relative purity) from SDS-PAGE gels shown in panels (red boxes) (**A**–**C**). (**E**) WB analysis confirming PV identity using anti-PV IgG antibody on the optimized extract (4 °C, 12 h, 1:6 ratio); β-actin (~42 kDa) is shown as the internal loading control for protein normalization. MW markers (M) are indicated on the left in kilodaltons (kDa).

Turbot PV was isolated from turbot muscle using a cold-buffered extraction protocol, followed by ammonium sulfate precipitation and dialysis, to obtain an enriched PV protein fraction preparation suitable for subsequent glycation and allergenicity studies. SDS-PAGE served as a key quality-control step to evaluate purity, MW, and integrity [34]. We demonstrate that optimal turbot PV extraction from fish muscle yields maximum yield and purity under specific buffer conditions (Figure 1).

The initial parameter optimized was temperature (Figure 1A). Densitometric analysis of SDS-PAGE bands showed that extraction at 0 °C resulted in the highest protein recovery (72,161), followed by 4 °C (67,822; 94% of 0 °C). In contrast, recovery declined at 8 °C (63,404; 88% of 0 °C) and at 12 °C (60,822; 84% of 0 °C), corresponding to reductions of 12% and 16%, respectively. Extraction time at 4 °C was tested using a 1:6 (*w*/*v*) solvent-to-sample ratio. The maximum PV yield observed at 12 h declined at longer durations (24–48 h) (Figure 1B). The solvent-to-sample ratio was optimized at 4 °C for 12 h, with 1:6 (*w*/*v*) providing the best extraction efficiency; higher ratios (1:10–1:18) did not significantly improve results (Figure 1C). The enriched PV protein fraction exhibited a major band around 14 kDa on SDS-PAGE (Figure 1D). To verify the semi-quantitative differences in IgE-binding capacity, WB analysis was performed, and the bands were specifically recognized by an anti-PV IgG antibody (Figure 1E). β-actin was used as an internal loading control to ensure equal protein loading across all lanes. These gentle extraction conditions effectively preserved antigenic epitopes and conformational integrity, enabling reliable analysis of subsequent structural and immunological modifications. The quality of this preparation supports meaningful research on how Maillard conjugation impacts allergenicity and bioactivity.

### 3.2. Synthesis and Characterization of CS–Sugar–PV Conjugates via the MR

#### 3.2.1. Spectrophotometric Analysis of MR Progression

**Figure 2:**
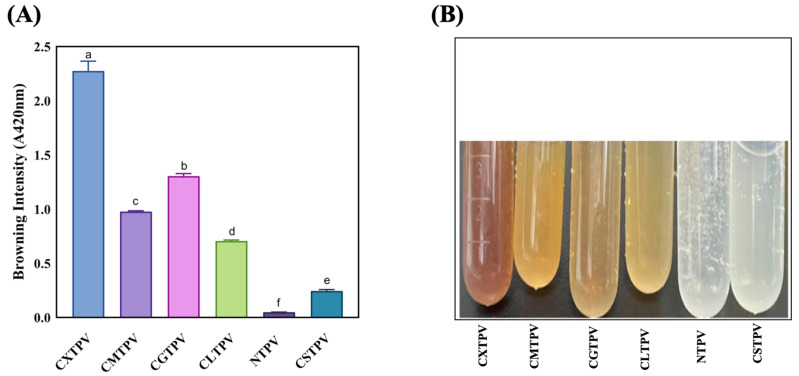
MR Browning of NTPV and CS–saccharide glycated conjugates. (**A**) Absorbance at 420 nm (A420) of NTPV and glycated conjugates (CXTPV, CGTPV, CMTPV, CLTPV, CSTPV). Data are presented as mean ± SD (n = 3). Different letters above bars denote significant differences (*p* < 0.05). (**B**) Photographs of the corresponding reaction solutions after 72 h incubation at 60 °C, illustrating color development.

Browning intensity (A420) is the most widely used, simple, and rapid method for monitoring non-enzymatic browning, reflecting the accumulation of high-MW melanoidins and advanced glycation end-products (AGEs) in the final stages of the MR [7]. The results revealed a clear sugar-dependent hierarchy among the conjugates: CXTPV (2.275 ± 0.093) > CGTPV (1.305 ± 0.022) > CMTPV (0.977 ± 0.007) > CLTPV (0.705 ± 0.010) > CSTPV (0.244 ± 0.013) (*p* < 0.05). The NTPV exhibited negligible browning (0.049 ± 0.001). All glycated conjugates showed significantly higher A420 values than NTPV (*p* < 0.05), with CXTPV exhibiting the highest absorbance (Figure 2A).

A visual examination of the reaction solutions after 72 h of incubation revealed a clear progression in color change, following the same hierarchy from no color change in NTPV to a deep brown in CXTPV (Figure 2B). The 46.4-fold increase observed in CXTPV highlights the high reactivity of the pentose sugar xylose. The very low NTPV value indicates that the browning effect was caused by CS–sugar-mediated glycation rather than heat treatment alone.

#### 3.2.2. Analysis of Glycation and Native Control by SDS-PAGE and WB

**Figure 3:**
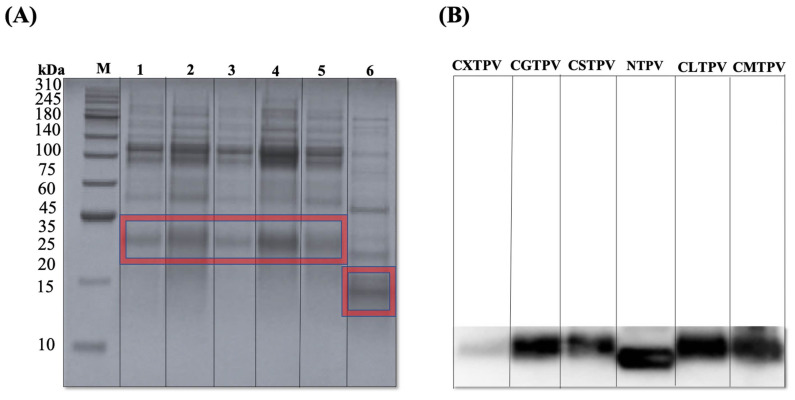
SDS-PAGE and WB analysis of NTPV and CS–saccharide glycated conjugates. (**A**) SDS-PAGE under reducing conditions. NTPV shows a sharp band at ~12 kDa, indicated by red boxes. Glycated conjugates show smeared, high-molecular-weight bands indicative of covalent attachment and polydispersity. Gel was stained with Coomassie Brilliant Blue. (**B**) Corresponding WB probed with anti-PV IgG antibody. NTPV exhibits immunoreactivity at ~14 kDa, while glycated conjugates show shifted (~20–25 kDa) immunoreactive bands at higher MW. MW markers (M) are indicated on the left in kilodaltons (kDa). 1: CGTPV, 2: CMTPV, 3: CXTPV, 4: CLTPV, 5: CSTPV, 6: NTPV.

MR forms covalent sugar adducts and cross-links, which appear on gels as high-MW smearing and reduced band intensity. These patterns provide direct visual proof of chemical conjugation and structural alteration, confirming that glycation leads to significant protein aggregation and denaturation [35]. SDS-PAGE analysis revealed a distinct MW shift for all glycated conjugates compared to the NTPV (Figure 3A), which migrated as a sharp band at approximately 14 kDa. The conjugates, CXTPV, CGTPV, CMTPV, CLTPV, and CSTPV, displayed smeared, higher-MW PV bands than those of NTPV.

The identities of the shifted bands were confirmed by Western blot with an anti-PV IgG antibody, which showed that the high-MW species contained PV (Figure 3B). The intensity and clarity of the IgG signal varied notably; for example, a heavily glycated conjugate such as CXTPV exhibited weaker, more diffuse reactivity. These structural modifications suggest that glycation with specific reducing sugars can markedly influence PV’s IgE- and IgG-binding abilities. Such conformational changes indicate that glycation, especially with sugars rich in reactive components like xylose, can substantially affect PV’s capacity to bind IgE and IgG.

### 3.3. Conformational Rearrangement of Glycated Conjugates and NTPV

#### 3.3.1. Evidence from CD

**Figure 4:**
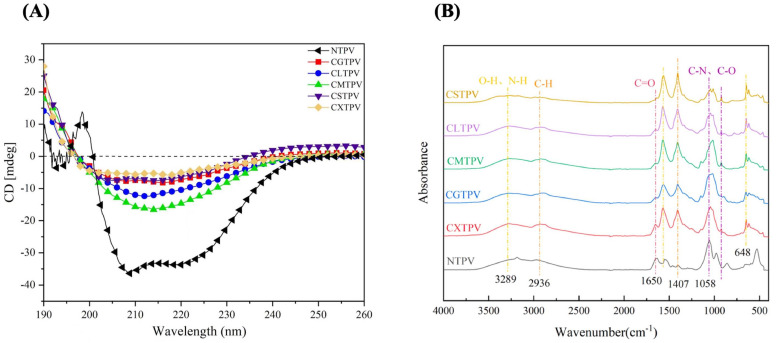
Structural changes in NTPV and CS–saccharide glycated conjugates assessed by spectroscopy. (**A**) Far-UV CD spectra. NTPV shows typical α-helical features (positive band ~198 nm; negative minima ~209 nm and 220–222 nm). Glycated conjugates exhibit reduced helical content and increased disordered structure. (**B**) FTIR spectra. Glycated conjugates display loss of the N-H stretch (~3188 cm^−1^) and modifications in the fingerprint region (1200–900 cm^−1^) relative to NTPV.

CD spectroscopy was used to quantitatively analyze changes in secondary structure in turbot PV induced by CS–saccharide glycation [36]. To understand how MR affects the PV structure of turbot, we examined the protein using CD spectroscopy. The spectral data (Figure 4A) showed that NTPV exhibits a typical α-helical pattern, with a positive peak between 195 and 200 nm, a maximum ellipticity at 198 nm, and two negative peaks at 209 and 220 nm. This spectrum displayed the classic α-helix signature with minima at 209 nm and 222 nm (Figure S1). Conversely, all glycated conjugates showed spectra indicating disordered and aggregated structures, characterized by a broad negative peak from 208 to 240 nm and a reduced positive peak from 190 to 208 nm (Figures S2–S6). The conjugates demonstrated a significant sugar-dependent decrease in α-helicity and an increase in β-sheet and random-coil content. This spectral shift indicates a glycation-induced conformational change from an ordered α-helical structure to one dominated by β-sheet and coil formations.

Quantitative analysis of the far-UV CD spectra revealed notable changes in PV’s secondary structure after CS–saccharide glycation (Table 1). NTPV showed an α-helix content of 29.67 ± 0.82% and a β-sheet content of 21.10 ± 0.62%. However, all glycated conjugates exhibited a highly significant decrease in α-helix content (*p* < 0.001), dropping from 6.16 ± 0.21% (CGTPV) to 2.05 ± 0.06% (CSTPV), along with a corresponding increase in β-sheet content, from 32.12 ± 0.96% (CGTPV) to 37.42 ± 1.10% (CSTPV). The greatest loss of α-helix and increase in β-sheet were observed in CSTPV and CXTPV, while levels of random coil and β-turn varied among the conjugates. This pattern confirms a consistent shift from helical to β-sheet-rich structures in all glycated samples compared to NTPV.

#### 3.3.2. Evidence from FTIR

FTIR spectroscopy primarily detects covalent bonds and functional groups, such as the loss of free amino groups and the emergence of Schiff bases or Amadori products, confirming the MR [37]. It verified covalent conjugation and sugar-dependent structural changes in CS–saccharide-PV conjugates (Figure 4B). NTPV showed characteristic protein bands: a broad O-H/N-H stretch at 3289 cm^−1^, C-H at 2936 cm^−1^, Amide I (C=O) at 1650 cm^−1^, Amide II (N-H bend) at 1564 cm^−1^, C-H bend at 1407 cm^−1^, C-N/C-O stretches at 1058 and 920 cm^−1^, C-H out-of-plane bend at 648 cm^−1^, and a peak at 529 cm^−1^ (Figure S7). The 529 cm^−1^ peak was absent in glycated samples, indicating disruption of higher-order structures or disulfide bonds. A sharp N-H band at 3188 cm^−1^ in NTPV disappeared in conjugates, implying N-H modification. The Amide II band at 1564 cm^−1^ increased, likely due to saccharide N-H groups. The band at 1407 cm^−1^ intensified, consistent with N-linked glycation or saccharide C–H addition and aligned with the increased intensity at 648 cm^−1^. The 1200–900 cm^−1^ region (C-O/C-N stretches) showed lower-wavenumber shifts, broadening, loss of a 976 cm^−1^ shoulder, and a new peak at 920 cm^−1^, indicating saccharide effects. CXTPV displayed prominent C=O and ~1050 cm^−1^ peaks (Figure S8). CGTPV and CMTPV exhibited broader C-O/C-N bands (Figures S9 and S10). CLTPV and CSTPV showed peak splitting in this region (Figures S11 and S12). CSTPV lacked a clear C=O band, suggesting extensive modification.

The relative proportions are shown in Table 2. Compared to the NTPV, the MR caused noticeable structural changes that varied with the saccharide. NTPV displayed a balanced secondary structure, with 29.1% α-helix, 27.9% β-turn, 24.3% random coil, and 18.9% β-sheet, which is typical of a well-folded calcium-binding protein. In contrast, glycation-driven reorganization: CGTPV, CMTPV, and CLTPV showed increased β-sheet content (22–25%) and decreased α-helix and β-turn levels. CSTPV led to the most significant disruption, with the highest random coil (31.86%) and the lowest α-helix content (18.26%), indicating severe unfolding. Interestingly, CXTPV had increased α-helical content (31.76%) and decreased β-sheet content, suggesting that xylose helped stabilize certain structural elements.

### 3.4. Free Amino Acid Analysis Reveals Sugar-Dependent Glycation Signatures

Free amino acids, especially lysine and arginine, act as the main nucleophiles initiating the MR by forming Schiff bases with reducing sugars [38]. Their covalent modification promotes protein cross-linking, structural changes, and the formation of advanced glycation end-products (AGEs), which directly impact functionality and allergenicity. Analysis of free amino acids revealed conjugate-specific changes compared to NTPV, providing direct evidence of MR progression beyond thermal effects (Table 3). Lysine consistently decreased across all glycated conjugates, with CXTPV showing the greatest reduction (56%), followed by CMTPV (50%) and CGTPV (43%), indicating the most extensive covalent modification. The CLTPV conjugate exhibited the most dramatic changes, with phenylalanine and tryptophan increasing by 800% each and histidine increasing by 554%, suggesting aggressive protein cross-linking and structural unfolding. Conversely, the CMTPV conjugate showed a unique 853% rise in glutamic acid, a hallmark of non-enzymatic deamination. The CGTPV conjugate displayed notable increases in aromatic and basic residues, with phenylalanine rising 453% and tryptophan by 473%, indicating AGE formation. The CSTPV and CXTPV conjugates exhibited more distinct profiles: CSTPV showed significant increases in glycine (131%) and arginine (114%), while CXTPV showed the highest overall increase in glycine (199%) along with marked lysine depletion.

### 3.5. Glycation Confers Potent Antioxidant Activity to PV Conjugates

**Figure 5:**
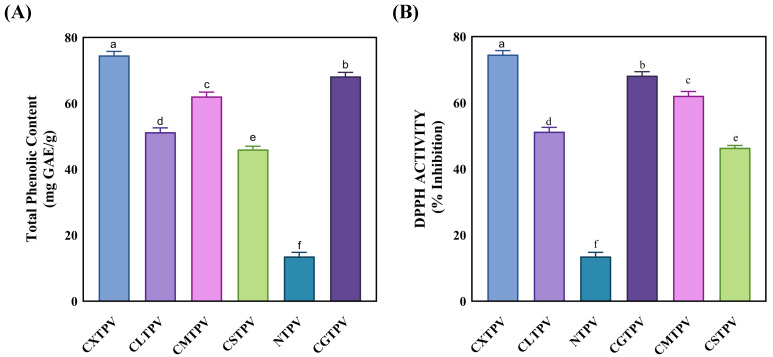
Antioxidant capacity NTPV and CS–saccharide glycated conjugates (CXTPV, CGTPV, CMTPV, CLTPV, CSTPV). (**A**) TPC in mg gallic acid equivalents (GAE) per g protein. All conjugates exhibited significantly higher TPC than NTPV (*p* < 0.05). Data are mean ± SD (n = 3). Different letters above bars denote significant differences (*p* < 0.05). (**B**) DPPH radical scavenging activity expressed as a percentage. All conjugates exhibited significantly higher activity than NTPV (*p* < 0.05). Data are mean ± SD (n = 3). Different letters above bars denote significant differences (*p* < 0.05).

A significant increase in TPC following glycation serves as a key functional biomarker, indicating that the MR concurrently attenuates allergenicity and confers enhanced antioxidant capacity, transforming the protein into a dual-action ingredient for hypoallergenic food applications [39,40]. TPC values (mg GAE/g protein) were markedly higher in all glycated conjugates compared to NTPV (5.4%) (Figure 5A). CXTPV showed the most substantial increase, reaching 10.3-fold higher than NTPV, while CSTPV, CGTPV, CLTPV, and CMTPV exhibited 6- to 9-fold elevations. This pronounced enhancement directly quantifies the abundance of reducing MRPs, which fulfill a dual role: as antioxidant species and as a covalent, steric matrix that disrupts conformational IgE-epitopes.

The DPPH assay is a standard method for measuring antioxidant activity by assessing free-radical scavenging [41]. The effect of glycation was evaluated by measuring both the antioxidant activity and total reducing capacity of the conjugates (Figure 5B). NTPV showed minimal activity (13.5%), indicating that heat treatment alone is insufficient. All CS-glycated conjugates demonstrated significant increases (*p* < 0.05), following a clear ranking: CXTPV (74.5%, 5.5-fold increase) > CGTPV (68.2%) > CMTPV (62.1%) > CLTPV (51.2%) > CSTPV (46.4%). The strong performance of CXTPV highlights the high reactivity of xylose (an aldopentose) in forming effective MRP when conjugated via the CS platform.

### 3.6. Immunological and Functional Profiling of Glycated PV Conjugates

#### 3.6.1. Indirect ELISA Reveals a Sugar-Dependent Reduction in IgE-Binding Capacity

**Figure 6:**
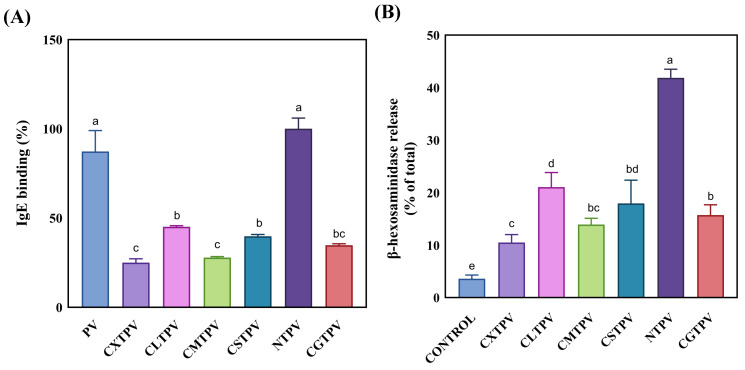
Reduction in IgE reactivity and mast cell degranulation by glycated PV conjugates. (**A**) Indirect ELISA showing IgE-binding capacity of NTPV and CS–saccharide conjugates (CXTPV, CMTPV, CGTPV, CSTPV, CLTPV). Values are expressed as mean ± SD (n = 3); all conjugates are significantly lower than NTPV (*p* < 0.001). Different letters denote significant differences (*p* < 0.05). (**B**) β-Hexosaminidase release assay in RBL-2H3 cells. Values are mean ± SD (n = 6); all conjugates are significantly lower than NTPV (*p* < 0.001). Different letters denote significant differences (*p* < 0.05). CONTROL: Tyrode’s buffer only treatment; PV (untreated).

ELISA is a widely used, highly sensitive immunological technique for measuring the specific binding of IgE antibodies to allergenic proteins. It enables the detection of both conformational and linear epitopes recognized by patient sera [42]. The IgE-binding capacity is a key indicator of sensitization potential and the presence of allergenic epitopes, serving as an essential initial assessment of the immunogenicity of PV-modified allergens [20]. In this study, indirect ELISA was employed to evaluate the IgE-binding capacity of NTPV and its CS–saccharide glycated conjugates. Untreated PV (Figure 6A) showed high IgE-binding levels (mean ± SD: 87.27 ± 11.72). NTPV exhibited a similarly high reactivity (100.0 ± 6.0), comparable to that of untreated PV. All CS–saccharide glycated conjugates displayed a significant reduction in IgE-binding capacity. A sugar-dependent effectiveness hierarchy was observed: CXTPV had the most potent suppression (24.97 ± 2.16), followed by CMTPV (27.77 ± 0.60), CGTPV (34.67 ± 0.85), CSTPV (39.66 ± 1.04), and CLTPV (45.03 ± 0.60). The leading conjugate, CXTPV, achieved approximately a 72% reduction in IgE binding compared to NTPV. These results suggest that thermal processing without reducing sugars does not decrease allergenicity.

#### 3.6.2. Mast Cell Degranulation Assays Confirm Functional Allergenicity Reduction

The measurement of β-hexosaminidase release is a reliable, quantitative, and widely recognized marker of mast cell degranulation and early allergic activation [43]. This well-validated in vitro model provides a sensitive and consistent measure of IgE-mediated mast cell activation, enabling direct evaluation of how glycation can diminish allergen-triggered degranulation, thereby reducing the elicitation phase of the allergic response. In this study, we used the β-hexosaminidase release assay with RBL-2H3 cells to assess the allergenic potential of NTPV and the CS–saccharide glycated conjugates. Stimulation with the NTPV process control caused a strong degranulation response, averaging 41.88 ± 1.84%. Conversely, all glycated conjugates significantly reduced this response (*p* < 0.001), as shown in Figure 6B. A clear hierarchy based on sugar dependency emerged, with CXTPV showing the strongest inhibition (10.30 ± 1.51%, about 75% decrease), followed by CMTPV (13.77 ± 1.25%, roughly 67% reduction), CGTPV (15.07 ± 1.82%, around 64% reduction), CSTPV (18.28 ± 4.35%, approximately 56% reduction), and CLTPV (21.07 ± 2.59%, about 50% reduction). This pattern of functional suppression matched the reduction in IgE-binding capacity observed via indirect ELISA. 

#### 3.6.3. Glycated Conjugates Modulate Cytokine Secretion, Suppressing Pro-Allergic Signals and Inducing Tolerogenic Profiles

**Figure 7:**
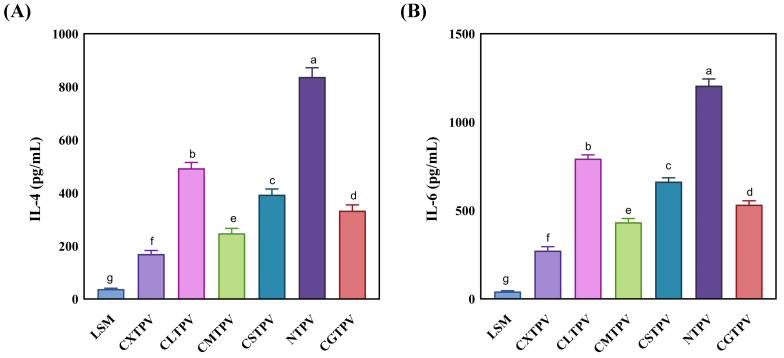
Effect of CS–saccharide glycation on cytokine secretion in RBL-2H3 mast cells stimulated with NTPV and conjugates. (**A**) IL-4 concentration in culture supernatants (pg/mL). Data are mean ± SD (n = 3). All conjugates significantly reduced IL-4 compared to NTPV (*p* < 0.001). Different letters above bars denote significant differences (*p* < 0.05). (**B**) IL-6 concentration in culture supernatants (pg/mL). Data are mean ± SD (n = 3). All conjugates significantly reduced IL-6 compared to NTPV (*p* < 0.001). Different letters above bars denote significant differences (*p* < 0.05). CONTROL: low-serum medium (LSM) treatment.

IL-4 is a pivotal Th2 cytokine that initiates and sustains allergic responses. It drives IgE production, Th2 cell differentiation, and mast cell activation, while amplifying broader pro-allergic cytokine networks. In food allergy, elevated IL-4 is directly linked to increased IgE, intestinal mast cell proliferation, heightened anaphylaxis, and a breakdown of immune tolerance [44]. To functionally assess the impact of glycation on allergic responses, the secretion of key cytokines (IL-4 and IL-6) by RBL-2H3 cells was quantified. This cell line is a well-established and validated model for studying IgE-mediated mucosal mast cell function and cytokine production in food allergy research [43]. Our work significantly demonstrates that the degree of immunomodulation is critically dependent on our CS–saccharide conjugation platform. The CS scaffold, with its high density of free amino groups, actively drives extensive covalent modification, creating a dense matrix of Maillard products on the protein surface [45]. This matrix facilitates the superior, sugar-dependent shielding of allergenic epitopes. Stimulation with the NTPV (Figure 7A) elicited a robust IL-4 response (837.5 ± 32.6 pg/mL). The robust IL-4 secretion from RBL-2H3 cells stimulated by NTPV signifies an adverse, pro-allergic response, as this cytokine is a master regulator that initiates and sustains the Th2-type immune response central to food allergy [46]. In stark contrast, all CS–saccharide glycated conjugates induced a profound and statistically significant (*p* < 0.001) reduction in IL-4 secretion. A clear, sugar-dependent hierarchy was observed: CXTPV was the most potent, reducing IL-4 levels by ~80%, from 170.0 ± 12.6 pg/mL to 34.0 ± 3.0 pg/mL. This was followed, in order of decreasing efficacy, by CMTPV, CGTPV, CSTPV, and CLTPV (~40% reduction).

IL-6 is a multifunctional pro-inflammatory cytokine rapidly secreted by activated mast cells, including the RBL-2H3 cell line, following IgE-mediated stimulation and degranulation. IL-6 plays a central role in amplifying allergic inflammation by promoting immune cell recruitment, enhancing Th2 polarization in sync with IL-4, increasing vascular permeability, and contributing to the maintenance of chronic allergic responses [47,48]. IL-6 secretion is recognized as a functional marker of mast cell activation and late-phase inflammatory signaling [49,50]. In this study, IL-6 secretion was quantified to evaluate the immunomodulatory effects of CS–saccharide glycation on PV. NTPV stimulation induced a robust IL-6 response (1206.7 ± 37.4 pg/mL). In contrast, all CS–saccharide glycated conjugates caused a profound and statistically significant reduction (*p* < 0.001) in IL-6 secretion (Figure 7B). A clear sugar-dependent hierarchy emerged: CXTPV (273.3 ± 21.1 pg/mL, ~77% suppression) > CMTPV (433.3 ± 21.1 pg/mL) > CGTPV (533.3 ± 21.1 pg/mL) > CSTPV (663.3 ± 21.1 pg/mL) > CLTPV (793.3 ± 21.1 pg/mL, ~34% reduction). The marked suppression of IL-6, most pronounced with CXTPV (~77% reduction), represents a highly beneficial anti-inflammatory and potentially anti-allergic outcome.

## 4. Discussion

MR-induced glycation has been receiving more and more attention as  a non-thermal technology for allergenic protein reduction, while adding functional properties such as antioxidant ability [14,42]. Our study extends this approach to turbot PV by employing CS as a macromolecular scaffold, conjugated with five different saccharides (xylose, glucose, maltose, lactose, and sucrose). Our findings reveal that CS–saccharide glycation significantly reduces IgE-binding activity and mast cell activation, while notably increasing antioxidant capacity. These findings support the hypothesis that this dual modification synergistically reduces allergenicity by masking epitopes and disrupting conformation. This occurs concurrently with the generation of bioactive MRPs that enhance antioxidant activity [20,21,51]. The optimized extraction conditions (4 °C, 12 h, 1:6 *w*/*v*) yielded an enriched PV extract that retained both the expected MW and size of approximately 14 kDa and immunoreactivity, as confirmed by SDS-PAGE and WB analyses, in line with optimized protocols that preserve the integrity and immunoglobulin-binding capacity of the PV allergen [21,22]. Maintaining this structural integrity is crucial for an accurate evaluation of processing-induced modifications (such as glycation) in allergenicity, permitting a consistent comparative evaluation of epitope masking or disruption without inducing extraction artefacts that might interfere with IgE-binding or cellular activation studies [34,52].

The saccharide-dependent hierarchy in browning intensity aligns with reported MR kinetics, as pentoses, such as xylose, exhibit faster enolization and dicarbonyl formation due to their higher proportion of reactive open-chain forms than hexoses or non-reducing disaccharides [53]. The significant increase in activity in the presence of CS further supports its catalytic role by providing many free amino groups that serve as better nucleophilic scaffolds, bringing reactants closer together to promote Schiff base formation and advance MRPs [39]. In sugar-based systems, CS has been shown to enhance glycation efficiency and promote MRP/melanoidin formation when acting as a reactive scaffold [20], thereby explaining the higher reactivity of CS–pentose conjugates. This connection between sugar chemistry and the nucleophilic microenvironment of CS offers an adjustable way to control glycation levels and MRP formation, sheds light on the differences observed among conjugates, and highlights the broader potential of polysaccharide-mediated Maillard conjugation to influence functional outcomes in processed food proteins.

Covalent conjugation was confirmed by SDS-PAGE and WB, showing higher-MW smearing and decreased band intensity. These results align with those of Tai et al. [3], who observed similar MW shifts and reduced immunoreactivity in microwave-assisted glycated mandarin fish PV, attributing these changes to sugar attachment and partial epitope masking. These findings support the idea that glycation induces structural changes that decrease antibody recognition, consistent with patterns observed in Maillard-bound food proteins, in which heterogeneous covalent adducts and cross-links hinder epitope accessibility and alter electrophoretic mobility [32,53]. Conversely, NTPV showed no significant MW increase, smearing, or band shifts on SDS-PAGE, consistent with PV’s known thermostability [54]. Heating at 60 °C generally preserves PV’s native conformation, as thermal denaturation is usually partial or reversible and does not typically involve covalent cross-linking or glycation. This structural stability suggests that glycation primarily impacts conformational and linear epitopes, reducing IgE/IgG binding in fish PV systems.

CD and FTIR analyses revealed a marked decrease in α-helical structures and an increase in β-sheet formations, indicating conformational rearrangement and denaturation caused by glycation. These findings align with those of Zhao et al. [54], who observed that Maillard modification of recombinant silver carp PV decreased α-helix content and increased β-sheet content, thereby reducing IgE/IgG binding. Huang et al. [9] also reported that glycation of Salangid icefish PV caused significant unfolding and loss of secondary structure compared to ultra-high-pressure treatment, supporting the idea that MR-induced denaturation disrupts conformational epitopes in fish PV. Free amino acid analysis emphasized lysine’s critical role as a nucleophile in MR initiation, consistent with its known reactivity with reducing sugars to form Schiff bases [38,55,56]. Changes in amino acid profiles further suggest sugar-dependent reaction pathways and additional modifications, as seen in earlier model studies [57,58]. Li et al. [59] demonstrated that different sugars lead to distinct patterns of AGE formation and reactivity in glucose–lysine–arginine Maillard models, highlighting the influence of saccharides on pathway selection. Liu et al. [57] showed that mutating lysine, arginine, and cysteine residues significantly affected IgE binding to processed shrimp allergens, underscoring the importance of specific amino acids in modulating the immune response. These results support the idea that glycation outcomes are primarily controlled by the chemical nature of the reducing sugar and the nucleophilic attack mechanisms involved, providing a mechanistic explanation for the variations among conjugates [59]. The reactivity specific to sugars not only causes structural changes but also results in the formation of various MRPs, which can affect both functionality and allergenicity in processed food proteins [56,58]. When thermal processing occurs without glycation, there are generally no significant changes in free amino acid levels, except for minor losses due to denaturation or oxidation. This is because reducing sugars do not nucleophilically attack lysine and arginine residues, a process that leads to Schiff base formation, Amadori rearrangement, and the patterns of depletion or buildup observed in actual Maillard conjugates [60,61].

The increased antioxidant-enhancing effect observed in MRPs in this study likely stems from their well-known ability to scavenge radicals and reduce oxidative stress, properties developed during glycation, including melanoidins [30,62]. Viturat et al. [51] noted higher antioxidant potential in CS–glucose Maillard adducts, attributed to MRP and melanoidin formation facilitated by the CS skeleton, which acts as a reactive site and helps maintain stable bioactive conformations. Similarly, Du et al. [30] found that MRPs derived from marine protein hydrolysates show strong DPPH radical-scavenging and phenolic properties, highlighting the role of advanced Maillard intermediates in overall antioxidant activity. These results emphasize that sugar-specific glycation pathways, particularly those mediated by polysaccharides such as CS, yield versatile MRPs with potent antioxidant properties [39,51]. This process occurs in various food systems, where the type of reducing sugar and the presence of amino groups influence MRP formation and radical-quenching ability, underscoring the multifunctionality of glycated proteins in processed foods [15,63]. On the other hand, thermal processing alone may not significantly boost antioxidant capacity [64]. Increased antioxidant activity in derivatives compared to native forms supports the idea that structural changes can improve bioactivity beyond what heat alone can achieve [62].

Our study’s immunological results align with established pathways showing how glycation influences allergenicity in food proteins. Glycation modifies lysine and nearby residues in IgE-binding epitopes, causing steric hindrance or structural changes that affect antibody recognition [7,36,65]. The NTPV context is crucial for understanding these effects. Since PV allergens are heat-stable, traditional thermal processing is ineffective at reducing their allergenicity because it cannot degrade the allergens’ structure or induce significant epitope disruption [3,65]. Heating alone does not form covalent bonds or cause the complex conformational shifts needed for hypoallergenicity, so IgE-binding epitopes remain functional and can still activate mast cells and Th2 responses. In contrast, CS–saccharide glycation achieves significant immunological reduction by covalently modifying the antigen and restructuring it, overcoming heat-treatment limitations. Wu et al. [21] previously demonstrated that glycosylating turbot PV significantly reduces IgE reactivity and inhibits Th2 cytokine production in KU812 basophils. This indicates that such modifications can interfere with both sensitization and effector stages of allergic reactions. The decreased release of β-hexosaminidase in RBL-2H3 cells further implies suppression of FcεRI cross-linking and mast cell degranulation, which are known to occur due to reduced epitope accessibility after glycation [53,66]. The lowered secretion of IL-4 and IL-6 aligns with reduced early activation and later inflammatory signaling, as glycosylated allergens tend to down-modulate Th2 polarization and inflammatory amplification [21]. Critically, this reduction in cytokine levels is protective rather than adverse. IL-4 drives IgE class switching and Th2 dominance [46,67], while excess IL-6 promotes inflammation, eosinophil recruitment, and chronic allergic response [67,68]. These effects mirror mechanisms used by effective anti-allergic therapies that target Th2 pathways or mast cells [44,49], supporting the idea that Maillard-type glycation could be a strategic approach to shift immunity away from pro-allergic responses.

In summary, our study shows that glycation can both weaken immediate effector responses and reduce downstream inflammatory amplification. This provides a dual immunological advantage beyond masking epitopes, marking a significant improvement over thermal treatment alone in reducing allergens.

## 5. Conclusions

This research demonstrates that the Maillard-induced conjugation of PV with CS and various saccharides significantly reduces its allergenicity while improving its functional properties. Structural analysis confirmed a successful covalent modification, marked by a decrease in alpha-helical content and a corresponding rise in disordered random coil fractions. These structural changes obscure clinical IgE-binding epitopes, leading to a 72% reduction in IgE binding and the suppression of pro-inflammatory mediators (IL-4, IL-6, and beta-hexosaminidase) in RBL-2H3 cell models. While domestic cooking often cannot eliminate the potent allergenicity of stable fish PVs, the 72 h dry-state incubation used here serves as a controlled model to investigate the potential of the MR under conditions that mimic the thermal stress and dehydration found in industrial processing. This study demonstrates, as a proof-of-concept, that CS-mediated glycosylation of a PV-enriched fraction can generate products with significantly reduced IgE-binding capacity. Developing such “hypoallergenic isolates” is a crucial step toward creating safe, functional ingredients for processed seafood products (such as surimi or condiments). Moreover, the significant reduction in immunoreactivity without compromising protein integrity suggests that these modified conjugates could serve as “hypoallergoids” for fish allergen immunotherapy. Unlike native allergens, which carry a high risk of anaphylaxis during clinical desensitization, these modified proteins offer a safer option for Oral and Sublingual Immunotherapy by preserving T-cell reactivity while reducing IgE-mediated responses. Future research should focus on in vivo validation and food-matrix sensory testing to verify the long-term stability and clinical safety of these conjugates. Specifically, exploring the inhibition of advanced glycation end-products will be vital to ensure these modified marine proteins meet international toxicological standards for human consumption and therapeutic applications.

## Figures and Tables

**Table 1:** Secondary structure composition (%) of NTPV and glycated PV conjugates (SD *n* = 3) derived from far-UV circular dichroism spectroscopy.

Sample	α-Helix	β-Sheet	β-Turn	Random Coil
NTPV	29.67 ± 0.82 ^a^	21.10 ± 0.62 ^a^	10.07 ± 0.27 ^a^	35.82 ± 0.99 ^a^
CGTPV	6.16 ± 0.21 ^b^	21.10 ± 0.62 ^a^	16.23 ± 0.46 ^b^	41.98 ± 1.09 ^b^
CLTPV	4.28 ± 0.11 ^c^	33.81 ± 0.98 ^c^	11.62 ± 0.30 ^c^	46.99 ± 1.44 ^c^
CMTPV	3.40 ± 0.09 ^cd^	33.81 ± 0.96 ^c^	13.17 ± 0.38 ^d^	46.28 ± 1.38 ^c^
CSTPV	2.05 ± 0.06 ^d^	37.42 ± 1.10 ^d^	14.96 ± 0.41 ^e^	40.94 ± 1.16 ^d^
CXTPV	2.21 ± 0.08 ^d^	36.94 ± 1.06 ^d^	14.24 ± 0.40 ^e^	42.61 ± 1.19 ^bd^

Note: Different lowercase letters in the same column indicate significant differences (*p* < 0.05) as determined by one-way ANOVA tests. Abbreviations used: NTPV: Native Turbot Parvalbumin; CGTPV: Chitosan Glucose Turbot Parvalbumin; CLTPV: Chitosan Lactose Turbot Parvalbumin; CMTPV: Chitosan Maltose Turbot Parvalbumin; CSTPV: Chitosan Sucrose Turbot Parvalbumin; CXTPV: Chitosan Xylose Turbot Parvalbumin.

**Table 2:** Secondary structure composition (%) of NTPV and CS–saccharide conjugated turbot PV (SD = 3) determined by FTIR spectroscopy.

Sample	α-Helix	β-Sheet	β-Turn	Random Coil
NTPV	29.06 ± 0.06 ^bc^	18.91 ± 0.07 ^e^	27.88 ± 0.04 ^a^	24.31 ± 0.05 ^c^
CGTPV	29.23 ± 0.04 ^b^	22.11 ± 0.04 ^d^	22.33 ± 0.04 ^f^	26.46 ± 0.06 ^b^
CLTPV	27.86 ± 0.04 ^d^	24.80 ± 0.04 ^b^	23.65 ± 0.05 ^d^	23.80 ± 0.03 ^d^
CMTPV	28.97 ± 0.04 ^c^	25.13 ± 0.05 ^a^	22.72 ± 0.06 ^e^	23.36 ± 0.06 ^e^
CSTPV	18.26 ± 0.06 ^e^	24.14 ± 0.04 ^c^	25.87 ± 0.04 ^c^	31.86 ± 0.04 ^a^
CXTPV	31.76 ± 0.04 ^a^	16.57 ± 0.06 ^f^	27.43 ± 0.03 ^b^	24.37 ± 0.05 ^c^

Note: Different lowercase letters in the same column indicate significant differences (*p* < 0.05) as determined by one-way ANOVA tests. Abbreviations used: NTPV: Native Turbot Parvalbumin; CGTPV: Chitosan Glucose Turbot Parvalbumin; CLTPV: Chitosan Lactose Turbot Parvalbumin; CMTPV: Chitosan Maltose Turbot Parvalbumin; CSTPV: Chitosan Sucrose Turbot Parvalbumin; CXTPV: Chitosan Xylose Turbot Parvalbumin.

**Table 3:** Free Amino Acid Concentration (mg/L, N = 3) in NTPV and CS-Glycated PV.

Free Amino Acids	NTPV	CGTPV	CSTPV	CXTPV	CLTPV	CMTPV
Phe	0.43 ± 0.04 ^a^	2.38 ± 0.05 ^b^	1.59 ± 0.04 ^c^	1.35 ± 0.05 ^c^	3.87 ± 0.06 ^d^	1.10 ± 0.04 ^e^
Tryp	0.15 ± 0.04 ^a^	0.86 ± 0.04 ^b^	0.24 ± 0.06 ^ac^	0.13 ± 0.04 ^a^	1.35 ± 0.04 ^d^	0.29 ± 0.04 ^c^
Leucine	0.96 ± 0.06 ^a^	1.48 ± 0.05 ^b^	0.97 ± 0.04 ^a^	2.06 ± 0.04 ^c^	2.50 ± 0.04 ^d^	0.73 ± 0.03 ^e^
Glycine	3.25 ± 0.06 ^a^	8.05 ± 0.04 ^b^	7.50 ± 0.04 ^c^	9.73 ± 0.04 ^d^	4.89 ± 0.04 ^e^	8.77 ± 0.05 ^f^
Threonine	0.14 ± 0.03 ^a^	0.66 ± 0.04 ^b^	0.30 ± 0.04 ^c^	0.26 ± 0.04 ^c^	0.77 ± 0.05 ^b^	0.35 ± 0.04 ^c^
Glutamic	0.26 ± 0.04 ^a^	0.56 ± 0.06 ^b^	0.66 ± 0.04 ^bc^	0.44 ± 0.04 ^ab^	0.96 ± 0.04 ^c^	2.43 ± 0.04 ^d^
Histidine	0.71 ± 0.04 ^a^	1.96 ± 0.04 ^b^	0.83 ± 0.04 ^a^	0.91 ± 0.04 ^a^	4.64 ± 0.04 ^c^	0.84 ± 0.04 ^a^
Arginine	1.18 ± 0.04 ^a^	2.86 ± 0.05 ^b^	2.53 ± 0.04 ^c^	1.77 ± 0.04 ^d^	2.48 ± 0.04 ^c^	2.41 ± 0.06 ^c^
Glutamine	1.62 ± 0.04 ^a^	1.82 ± 0.04 ^b^	1.85 ± 0.04 ^b^	1.03 ± 0.04 ^c^	1.46 ± 0.04 ^a^	1.12 ± 0.04 ^c^
Lysine	2.36 ± 0.06 ^a^	1.34 ± 0.04 ^b^	1.56 ± 0.04 ^c^	1.03 ± 0.04 ^d^	1.46 ± 0.04 ^c^	1.19 ± 0.06 ^d^
Ornithine	2.27 ± 0.04 ^a^	3.82 ± 0.06 ^b^	2.63 ± 0.04 ^c^	1.65 ± 0.04 ^d^	4.22 ± 0.06 ^e^	1.87 ± 0.04 ^f^

Note: Different lowercase letters in the same column indicate significant differences (*p* < 0.05) as determined by one-way ANOVA tests. Abbreviations used: NTPV: Native Turbot Parvalbumin; CGTPV: Chitosan Glucose Turbot Parvalbumin; CLTPV: Chitosan Lactose Turbot Parvalbumin; CMTPV: Chitosan Maltose Turbot Parvalbumin; CSTPV: Chitosan Sucrose Turbot Parvalbumin; CXTPV: Chitosan Xylose Turbot Parvalbumin, Phe: Phenylalanine; Tryp: Tryptophan.

## Data Availability

The original contributions of this study are included in the article/Supplementary Material. Further enquiries can be directed to the corresponding author.

## Supplementary Materials

The following supporting information can be downloaded at: https://www.mdpi.com/article/10.3390/foods15071259/s1, Figure S1: Circular dichroism spectrum of CGTPV. Figure S2: Circular dichroism spectrum of CLTPV. Figure S3: Individual Circular dichroism spectrum of CMTPV. Figure S4: Circular dichroism spectrum of CSTPV. Figure S5: Circular dichroism spectrum of CXTPV. Figure S6: Circular dichroism spectrum of NTPV. Figure S7: Individual FTIR spectrum of Native turbot Parvalbumin, NTPV. Figure S8: Individual FTIR spectrum of turbot Parvalbumin glycated with chitosan-Xylose conjugate (CXTPV). Figure S9: Individual FTIR spectrum of turbot Parvalbumin glycated with chitosan-glucose conjugate (CGTPV). Figure S10: Individual FTIR spectrum of turbot Parvalbumin glycated with chitosan-maltose conjugate (CMTPV). Figure S11: Individual FTIR spectrum of turbot Parvalbumin glycated with chitosan-lactose conjugate (CLTPV). Figure S12: Individual FTIR spectrum of turbot Parvalbumin glycated with chitosan-sucrose conjugate (CSTPV).

## Abbreviations

The following abbreviations are used in this manuscript:

PV	Parvalbumin
MR	Maillard Reaction
ELISA	Enzyme-linked Immunosorbent Assay
MW	Molecular weight
DPPH	2,2-diphenyl-1-picrylhydrazyl
TPC	Total Phenolic Content
IgE	Immunoglobulin E
FTIR	Fourier-Transform Infrared
CD	Circular Dichroism
SDS-PAGE	Sodium Dodecyl Sulfate-Polyacrylamide Gel Electrophoresis
CS	Chitosan
HRP	Horseradish peroxidase
IL-4	Interleukin-4
IL-6	Interleukin-6
RBL-2H3	Rat Basophilic Leukemia
WB	Western Blot
MRPs	Maillard Reaction Products
PBS	Phosphate-Buffered Saline
FBS	Fetal Bovine Serum
BSA	Bovine Serum Albumin
DMEM	Dulbecco’s Modified Eagle’s Medium
KBr	Potassium bromide
BeStSel	Beta Structure Selection
LC-MS	Liquid Chromatography Tandem-Mass Spectrometry
IgG	Immunoglobulin G
Phe	Phenylalanine
Tryp	Tryptophan
TMB	3,3′,5,5-Tetramethylbenzidine
kDa	Kilodaltons
CXTPV	Chitosan xylose Turbot parvalbumin
CGTPV	Chitosan glucose Turbot Parvalbumin
CMTPV	Chitosan maltose Turbot Parvalbumin
CSTPV	Chitosan Sucrose Turbot Parvalbumin
CLTPV	Chitosan lactose Turbot Parvalbumin
NTPV	Native Turbot Parvalbumin
AGE	Advanced Glycation End-products
EDTA	Ethylenediaminetetraacetic acid
Tris-HCL	Tris(hydroxymethyl)aminomethane hydrochloride
GAE	Gallic acid equivalents
LSM	Low serum medium
