# Supplementary material for: Optimizing the Functional and Safety Properties of a Marine Allergen: Maillard-Induced Conjugation of Chitosan and Saccharides Attenuates the Allergenicity of Turbot (Scophthalmus maximus) Parvalbumin
Source: Foods. 2026 Apr 7;15(7):1259. doi: 10.3390/foods15071259 (PMC13073560; doi:10.3390/foods15071259)
Supplement: Supplementary file 1 [file foods-15-01259-s001.zip › foods-4183354-supplementary.pdf]

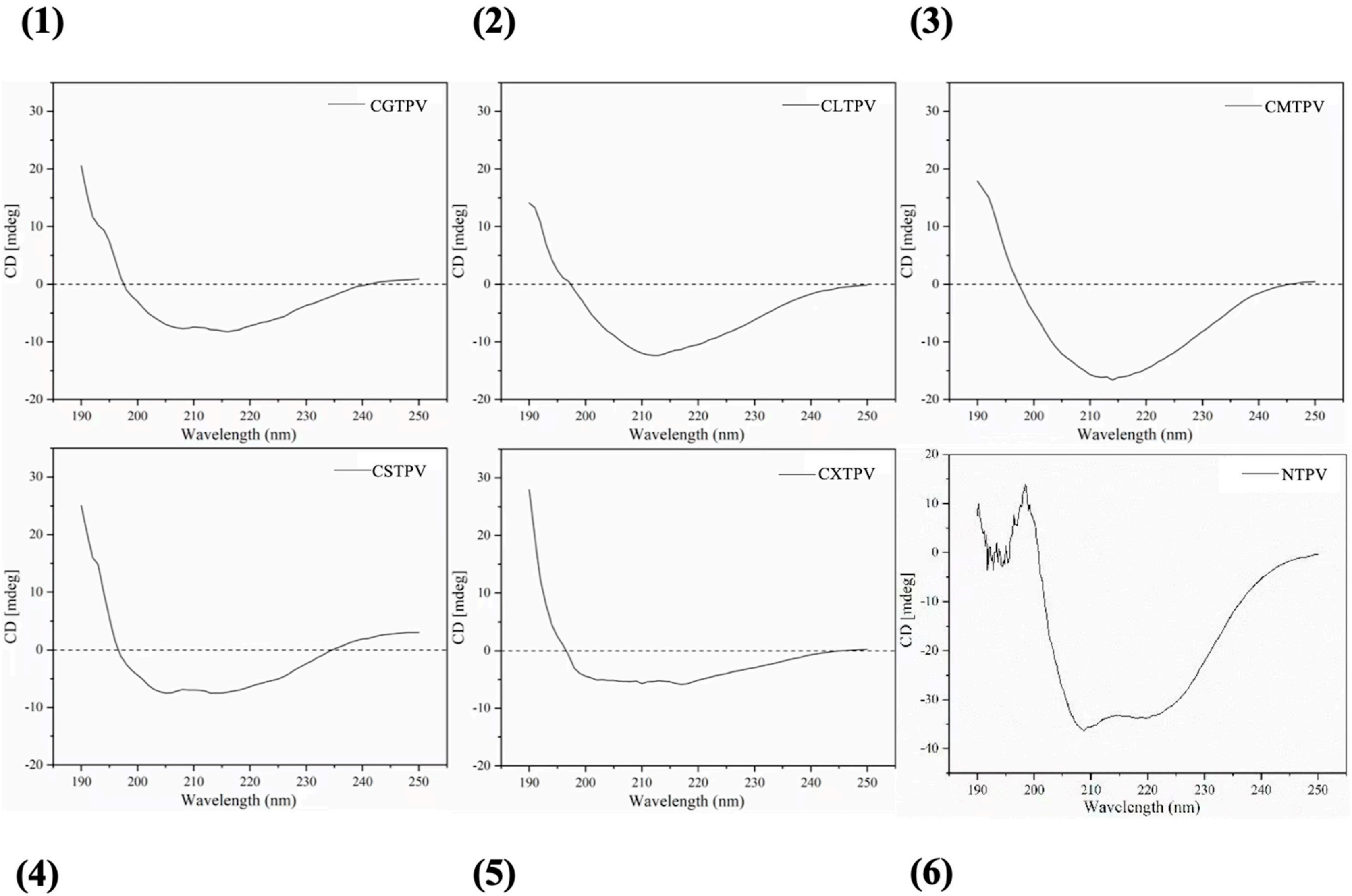
(1)
(2)
(3)
CGTPV
CLTPV
CMTPV
CSTPV
CXTPV
NTPV
CD [mdeg]
Wavelength (nm)
30
20
10
0
-10
-20
-30
-40
190
200
210
220
230
240
250
(4)
(5)
(6)

Figure S1: Circular dichroism spectrum of CGTPV. The spectrum exhibits a characteristic α-helical profile with a positive peak at ~198 nm and negative minima at 209 nm and 222 nm.

Figure S2: Circular dichroism spectrum of CLTPV. The spectrum exhibits a positive peak in the 190-208 nm range and a continuous negative peak in the 208-240 nm range.

Figure S3: Individual Circular dichroism spectrum of CMTPV. The spectrum exhibits a positive peak in the 190-208 nm range and a continuous negative peak in the 208-240 nm range.

Figure S4: Circular dichroism spectrum of CSTPV. The spectrum exhibits a positive peak in the 190-208 nm range and a continuous negative peak in the 208-240 nm range.

Figure S5: Circular dichroism spectrum of CXTPV. The spectrum exhibits a positive peak in the 190-208 nm range and a continuous negative peak in the 208-240 nm range.

Figure S6: Circular dichroism spectrum of NTPV. The spectrum exhibits a positive peak in the 190-208 nm range and a continuous negative peak in the 208-240 nm range.

(7)

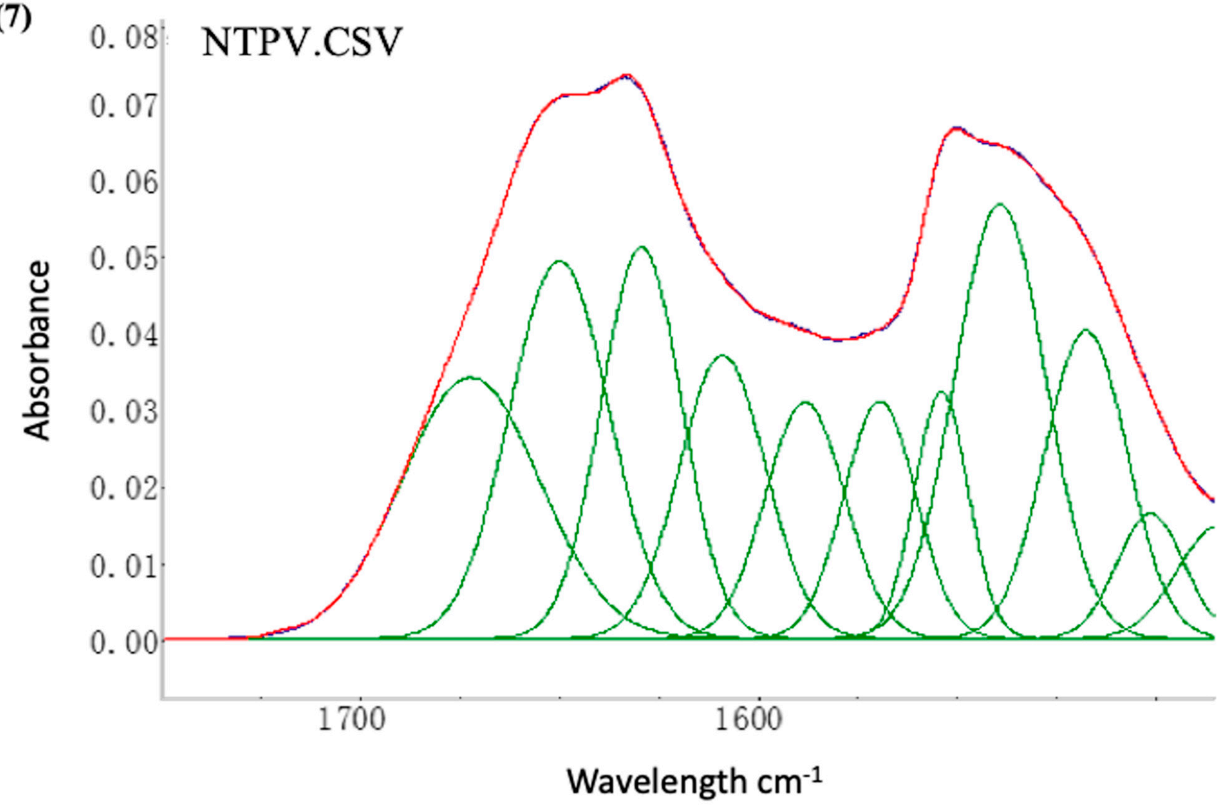

Figure S7: Individual FTIR spectrum of Native turbot Parvalbumin, NTPV.

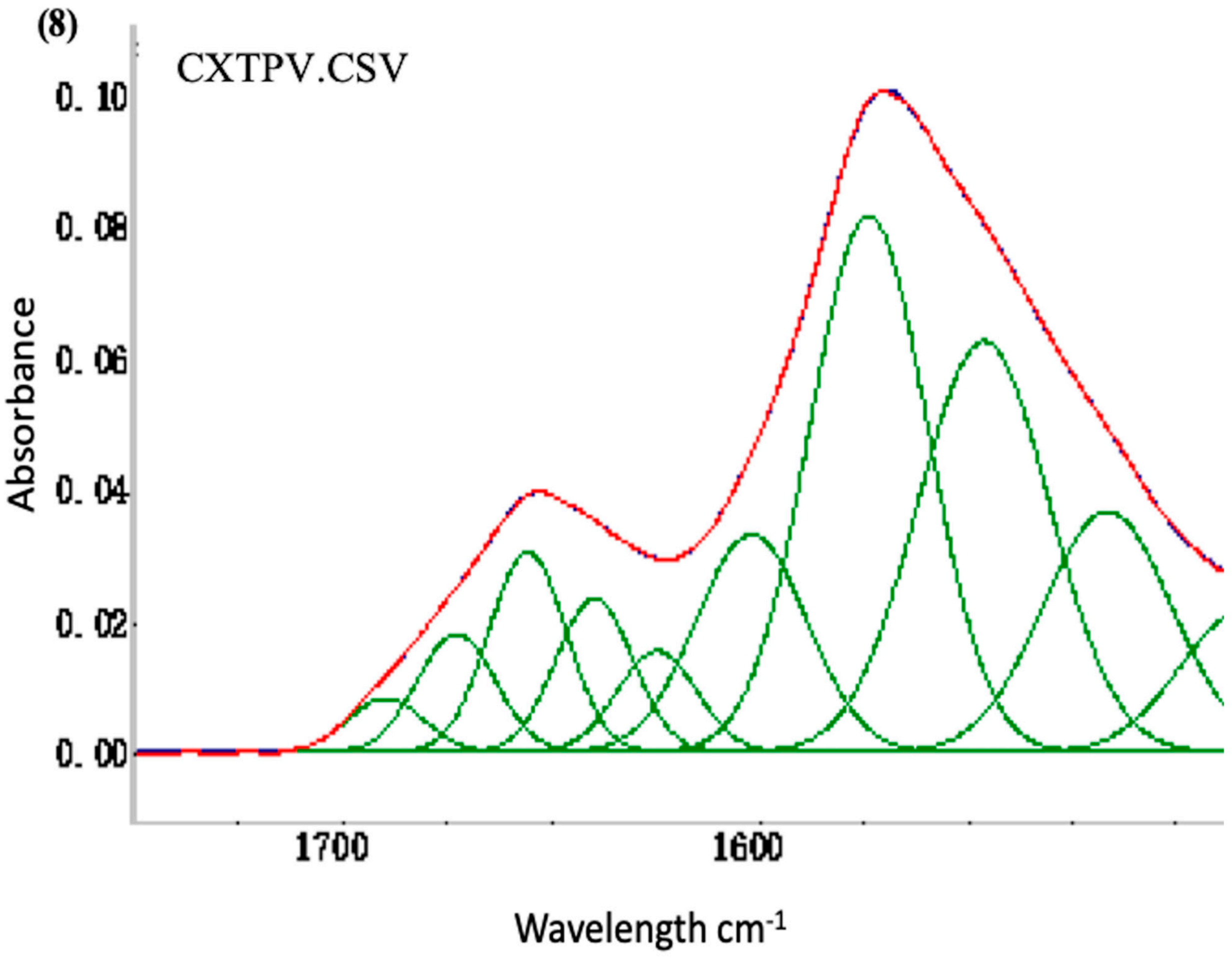

Figure S8: Individual FTIR spectrum of turbot Parvalbumin glycated with chitosan-Xylose conjugate (CXTPV).

(9)

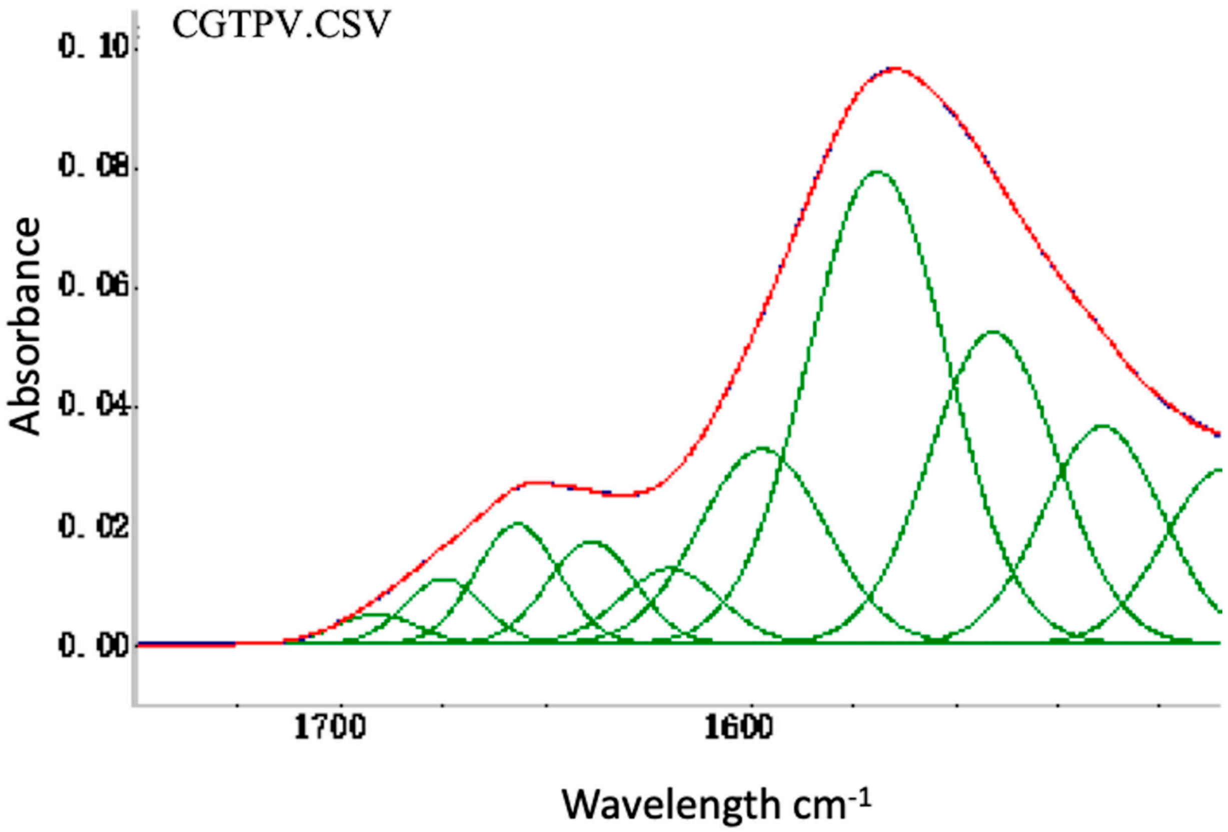

Figure S9: Individual FTIR spectrum of turbot Parvalbumin glycated with chitosan-glucose conjugate (CGTPV).

(10)

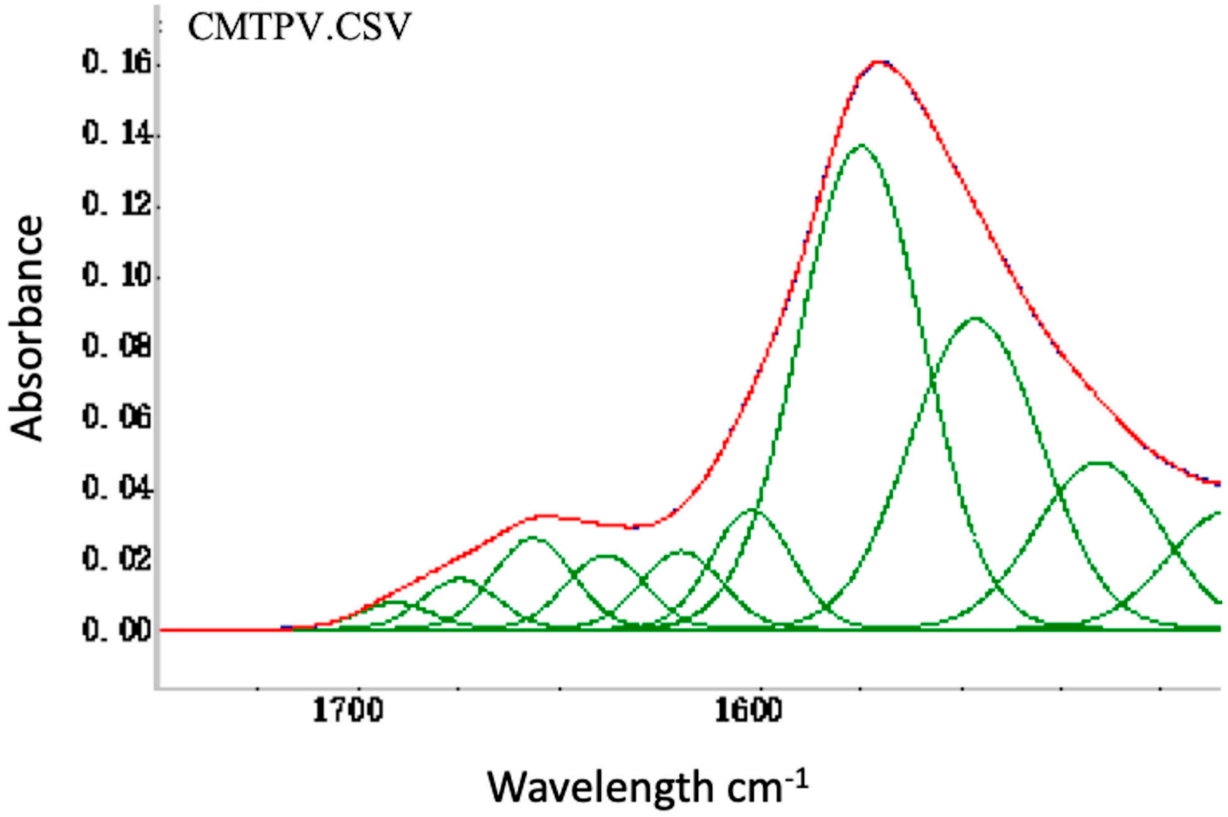

Figure S10: Individual FTIR spectrum of turbot Parvalbumin glycated with chitosan-maltose conjugate (CMTPV)

(11)

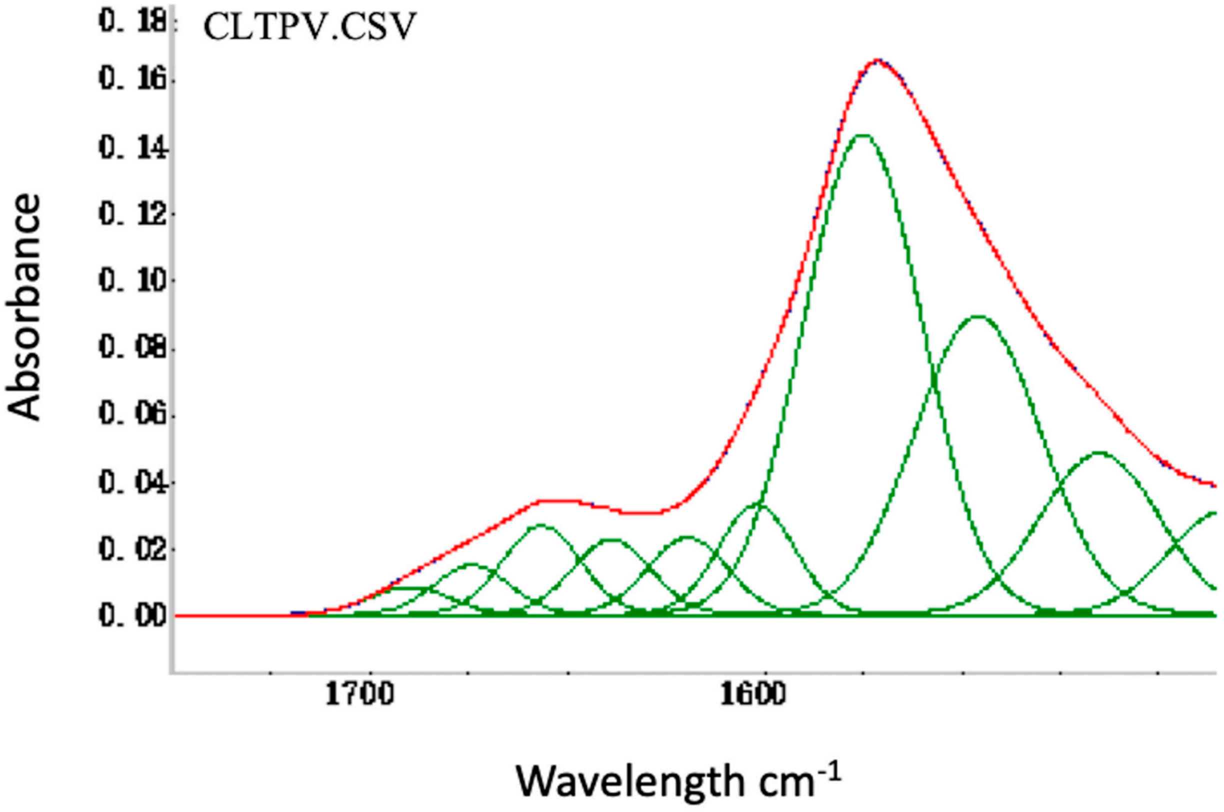

Figure S11: Individual FTIR spectrum of turbot Parvalbumin glycated with chitosan-lactose conjugate (CLTPV).

(12)

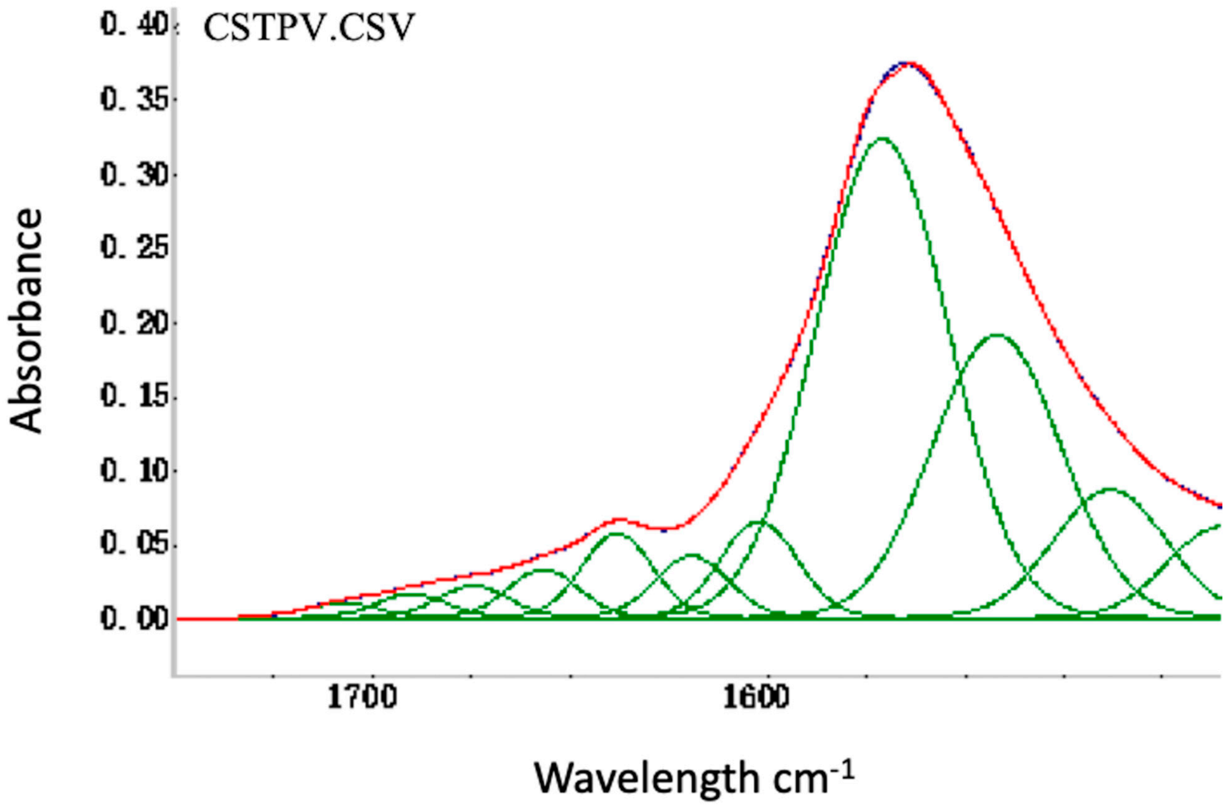

Figure S12: Individual FTIR spectrum of turbot Parvalbumin glycated with chitosan-sucrose conjugate (CSTPV).
